# Population Sex Ratios: Another Consideration in the Reintroduction – Reinforcement Debate?

**DOI:** 10.1371/journal.pone.0075821

**Published:** 2013-09-26

**Authors:** Sergio A. Lambertucci, Martina Carrete, Karina L. Speziale, Fernando Hiraldo, José Antonio Donázar

**Affiliations:** 1 Laboratorio Ecotono, INIBIOMA (CONICET-Universidad Nacional del Comahue), Bariloche, Argentina; 2 Department of Physical, Chemical and Natural Systems, University Pablo de Olavide, Seville, Spain; 3 Department of Conservation Biology, Estación Biológica de Doñana, CSIC, Seville, Spain; Bangor University, United Kingdom

## Abstract

Reintroduction or reinforcement (RorR) of wild populations is a common conservation strategy. Many conservation projects involve the release of individuals of poorly studied species. This may lead to inefficient results or negative impacts on the conservation efforts. Here, we provide new insights into the conservation implications and potential consequences of a skew in the sex ratio of released birds and of the number of birds supplemented for the demography of a long-lived dimorphic bird species, the Andean condor ( *Vultur* *gryphus* ). We demonstrate that a RorR conservation program may be less effective in conserving a species if the sex ratios of the releases and the recipient populations are not considered. We also show that releases can reduce population declines but only if carried out over long periods (i.e., several decades). This can mean high costs for release programs and the added challenge of maintaining programs over time. If RorR programs are to be implemented, bearing in mind the importance of properly assessing their effectiveness, we urge conservation researchers and managers to consider the implications of sex ratio biases for wild populations, and particularly for dimorphic species with sexually despotic behaviour.

## Introduction

Reintroduction or reinforcement (RorR) of wild endangered populations is typically advised after other conservation actions have been applied, and when most of the limiting factors involved have been mitigated or removed [1]. The latter issue is particularly important as RorR should be considered a last resort, implemented only after environmental causes of decline have been dealt with [2]. To guarantee its suitability and success, RorR must be based on in-depth studies of the biology and ecology of the species and its habitat, following common assessment criteria [1–3]. Despite these concerns, RorR approaches are very often among the first attempts aiming to overcome species decline [2–4]. In fact, many projects do not even address basic criteria proposed for RorR implementation, and thus programs are not carefully planned and conservation objectives are not met [3–5].

A lack of information on the biology of the species to be released is a common shortcoming frequently hindering the success of RorR strategies [2,3]. Negative impacts imposed by low short-term population fitness due to inbreeding or outbreeding depression, alteration of demographic parameters and introduction of diseases and pathogens, are frequently overlooked [1,3]. For instance, RorR programs sometimes fail to account for the importance of basic demographic assumptions, which may have unexpected consequences for the species meant to be protected [4]. In many cases individuals are released only according to their availability in source captive breeding programs and rehabilitation centres.

It is known that releasing individuals of different age classes can influence the demography of a population [4]. The consequences of releasing different sexes, however, are not clear. In sexually monomorphic species there may be an absence of sex bias at birth, mortality and dispersal allowing the release of both sexes at random [5]. However, this may not be the case for dimorphic species given that any of those demographic parameters may be asymmetric between the sexes. Therefore, we analyse the effects of different current and hypothetical reinforcement scenarios on a threatened long-lived species with strong sexual dimorphism, the Andean condor ( *Vultur* *gryphus* ). The sex ratios of wild populations are strongly male-skewed with age throughout the entire distribution range [6]. RorR programs for this species seek to maintain viable populations by releasing rehabilitated and captive-bred individuals into the wild [7]. However, the sex ratio of the releases and that of the recipient populations have not yet been considered. In fact, more male condors are released in some cases [6]. This male-skewed sex ratio could affect the demographic prospects of the species, although no attention has been paid to these potential consequences.

**Figure 1:**
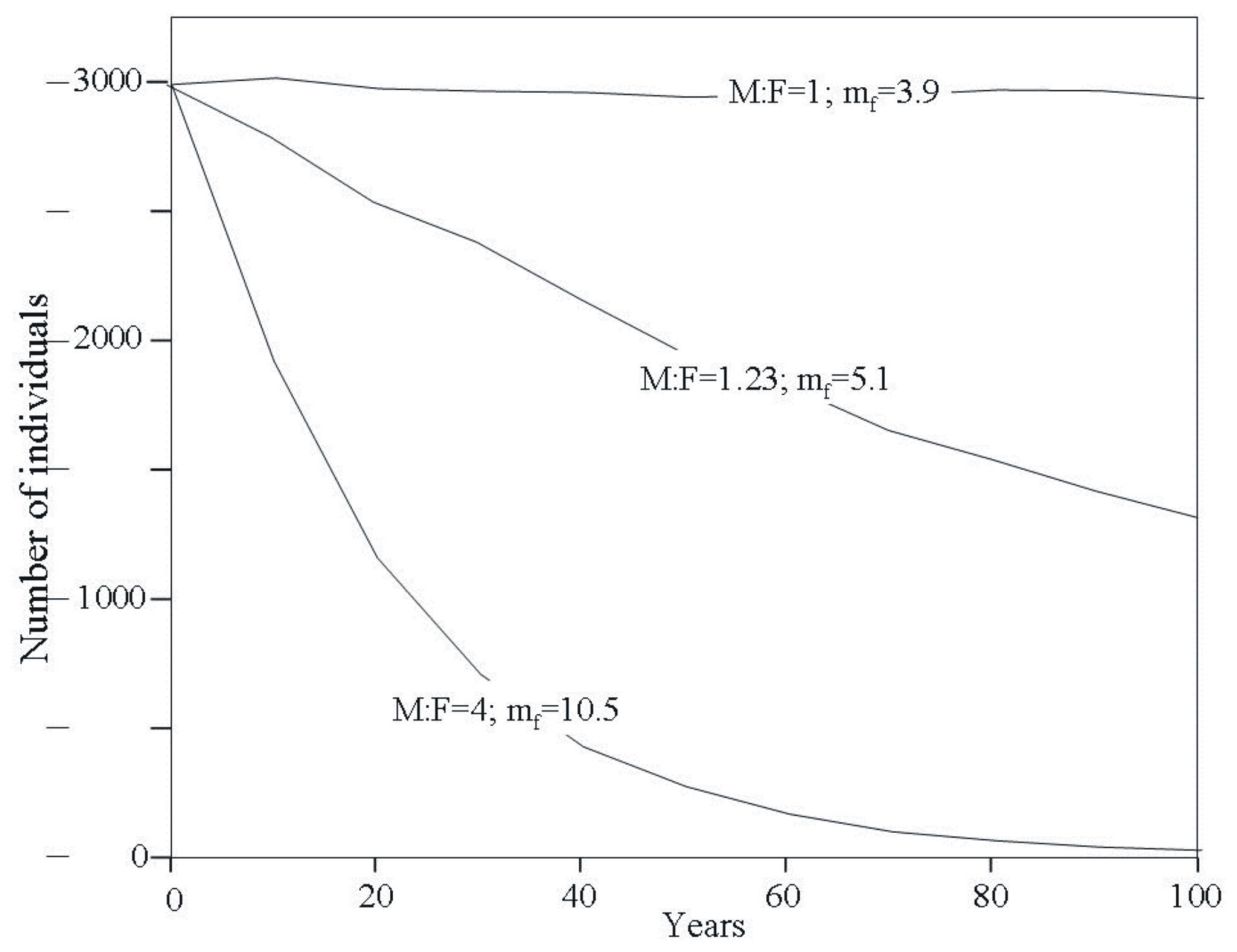
Prospective trajectories of a population of Andean condors when considering different adult sex ratios projected through increases in adult female mortality. Lines are mean values of the stochastic runs for each time step. Adult female mortality (m_f_) and adult male:female ratio (M:F) are shown for their corresponding population trajectories.

In this paper, we model the effects of releasing different sex ratios and different numbers of individuals into wild populations to consider their possible impact on the Andean condor populations. Specifically, we analyse the effects of actual and hypothetical reinforcement scenarios on the population viability of the species. We hypothesise that both the sex and the number of released birds influence the demographic response of recipient populations of sexually dimorphic species. We expect that releasing more individuals over time would positively influence population numbers, but that the best demographic results would be obtained if both the number and the sexual composition of the birds are considered. In particular, and considering that the populations of condors are male-biased, we expect that releasing more males would be less effective than releasing more females. To address these questions, we used simulation models (Population Viability Analysis, PVA [8]) to identify changes in population outcomes given different RorR scenarios. Very importantly, in this paper we will not evaluate the current state or trends of condor populations, but rather present a theoretical approach to demonstrate the possible effects of not considering the sex ratios of released birds.

**Figure 2:**
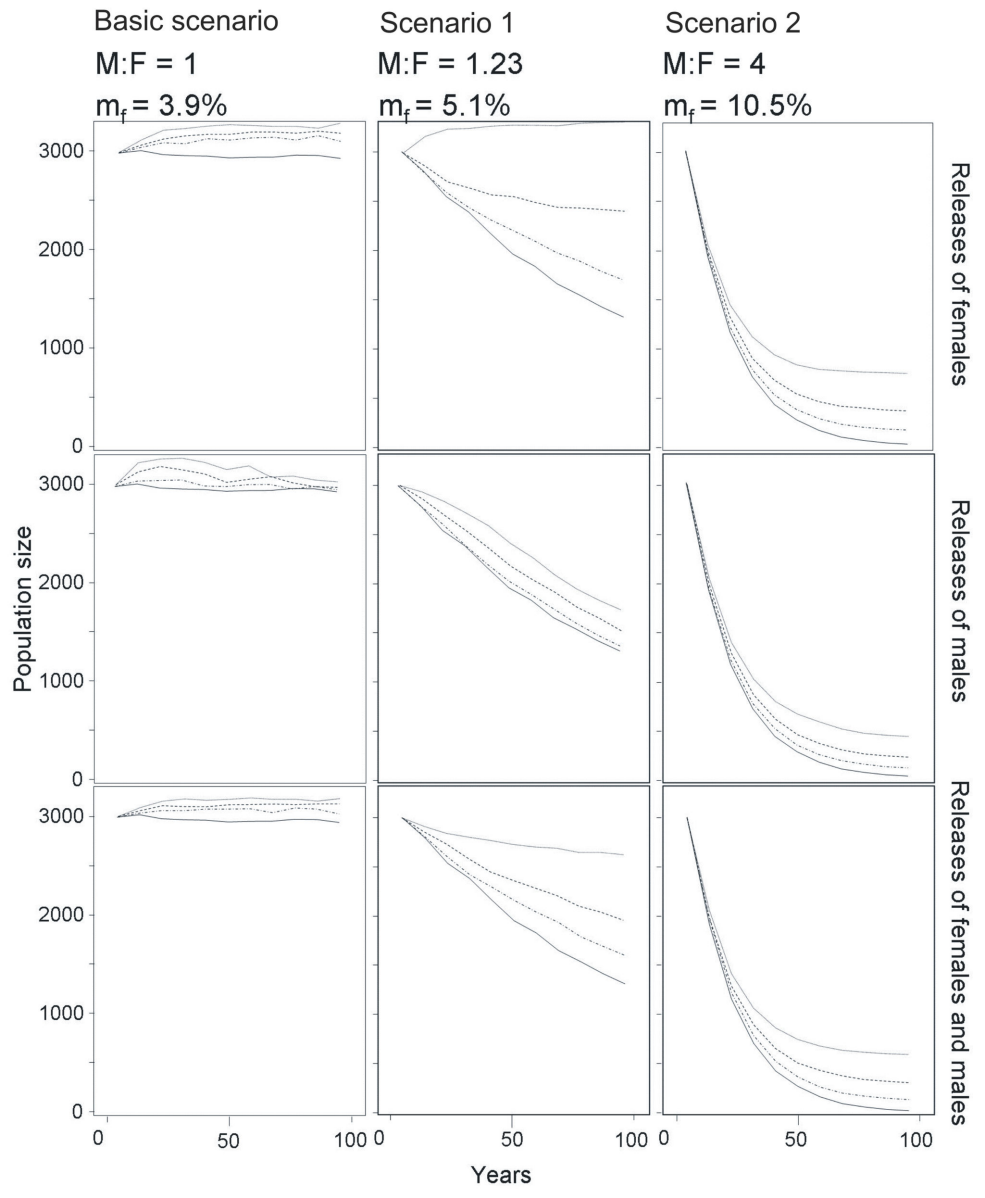
Projections of Andean condor population size over time under different reinforcement scenarios. Current reinforcement scheme using the mean number of birds reintroduced in Colombia between 1989-2005 (4.06 birds/year, dotted-dashed line), and two hypothetical reinforcement schemes (10 birds/year, dashed line; 20 birds/year, light solid line). Thick solid line represents the basic scheme without releases. Each column of plots shows the three scenarios of recipient populations with different sex ratios.

## Methods

### Study species

The Andean condor is considered near-threatened worldwide because of the reduction in its abundance and distribution over the last century [7]. It has become nearly extinct in its Northern range (i.e., Colombia and Venezuela), and population numbers are suffering throughout its distribution [9,10]. In this sexually dimorphic species, males are up to 30% larger than females, and have a despotic behavior with a marked hierarchy related to sex and age, with male adults at the top and juvenile females at the lowest rank [11]. This produces a segregation in the use of space and might favor a sex-biased mortality toward females [6,11,12]. Andean condors are long-lived birds, with very low reproductive rates (breeding pairs rear one chick every other year) [13,14].

### Population Viability Models and Analyses

We explored the possible influence of biased sex-ratios, both of wild recipient populations and of the reintroduced stocks, on the population viability of Andean condors by using the program VORTEX V9, a widely used individual-based simulation model for PVA [8,15]. We fitted a basic scenario using the demographic parameters known for the Andean condors, and considering their particular reproductive cycle of one egg every other year and a sex ratio of 1:1 [13,14,16].

Age of first breeding was set at 6 years since this is the age of sexual maturity and first laying observed in captivity and in wild birds [16,17] (authors unpublished data). Maximum age of reproduction (55 yrs) corresponds to the age observed for an Andean condor in captivity at the National Zoo, Washington D.C. [18]. Those values are optimistic demographic scenarios, since condor populations may first breed at higher ages and the maximum reproduction may be at lower rates [19]. Mortality rates of this species are not well-known, with only a local estimate available that is difficult to extrapolate to other populations within the entire condor range [20]. Moreover, those estimations lead to population declines even in the absence of a sex-bias (data not shown). Thus, we used a theoretical mortality rate to begin simulations with a stable population, without biased sex ratios, as a control against which to contrast different scenarios. However to be as realistic as possible we used the mortality rates of a wild population of a similar species, the bearded vultures ( *Gypaetus* *barbatus* ) breeding in the Spanish Pyrenees [21] (Table 1). This species is also a large, long-lived vulture, with delayed sexual maturity, and low reproductive rates, that breeds solitarily in mountain cliffs, much like condors [9,13,22]. These mortality values should be considered a theoretical starting point, as mortality rate values are not taken from condors. However, as the aim of this article is to demonstrate the consequences of sex-biased releases for populations of dimorphic species and not the demographic prospects of the Andean condor, they are appropriate for modelling purposes. We considered that released birds have the same mortality and breed at the same rate as wild ones for our modelling purposes, which is a conservative assumption.

**Table 1:** Demographic parameters used to simulate population trends.

Parameter	Basic Scenario (± SD)	Scenario 1 (± SD)	Scenario 2 (± SD)
Age of first offspring	6 years	6 years	6 years
Maximum age of reproduction	55 years	55 years	55 years
% adult female breeding successfully	50 (5)	50 (5)	50 (5)
% of broods with 1 offspring	50	50	50
% of female mortality from age 0 to 5	5.6 (1)	5.6 (1)	5.6 (1)
% of male mortality from age 0 to 5	5.6 (1)	5.6 (1)	5.6 (1)
% of female mortality after age 6	3.9 (1)	5.1 (1)	10.5 (1)
% of male mortality after age 6	3.9 (1)	3.9 (1)	3.9 (1)
Adult M:F ratio	1	1.23	4

Initial population size was arbitrarily fixed at 3,000 birds, without setting a carrying capacity for the study system. M:F= proportion of males to females. Mortality rates are theoretical and the basic scenario was based on a healthy population of a similar species (see [21,22] and methods).

Starting from our basic scenario (proportion of males to females (M:F)=1), we first increased female mortality to run alternative scenarios of skewed adult sex ratios corresponding to the minimum (M:F= 1.23: population sex-ratio scenario 1) and maximum (M:F = 4: population sex-ratio scenario 2) values of sex-ratio skewness observed in wild populations of condors throughout their distribution range [6] (Table 1).

Models were subsequently re-fitted to simulate the demographic consequences of current RorR programs releasing equal numbers of males and females, as well as of sex-biased reinforcements, which were implemented by supplementing different proportions of immature individuals of both sexes. We used information on condor reinforcements performed during 1989-2005 in Colombia, where 36 immature males and 29 immature females were released in total [23]. We used the mean rate of reinforcements per sex, calculated from these data obtaining a current supplementation scenario of 2.25 males and 1.81 females per year. To analyse the outcome of releasing the same number of individuals, but all of one sex, we added both values obtaining a supplementation scenario of 4.06 females or males per year. Finally, we simulated larger supplementations (10 and 20 individuals per year) to estimate whether those larger releases could help to correct negative population trends. Simulations were set to start from a population of 3000 individuals (almost half of the entire mature population suggested for the species [7]), and over a long period of time (100 yrs). For comparisons we also presented the differences in population trends when starting from subpopulations such as the one existing in the north-western region of Patagonia, Argentina (300 individuals [24]) and the number of birds that may be flying in Venezuela (30 individuals [7]).

We tested differences between scenarios through generalized linear mixed models (glmm), with the number of individuals (log-transformed) per year (n=100) as a dependent variable (normal error distribution, identity link function), and scenarios (i.e., different female mortalities -and thus sex ratio skewness, and different reinforcement schemes) and years as explanatory variables. To control for non-independence, we included runs (n=100) as a random factor in glmm. As population sizes were correlated over years, we considered a first-order autoregressive covariance structure. Models were performed using SAS 9.2 with a Laplace approximation and a between-within method for computing the denominator degrees of freedom.

## Results

In our basic scenario -in which female annual mortality is the lowest and equals that of males (3.9%)- and starting from a population of 3000 individuals, the population size of Andean condors was stable (Table 1; Figure 1). Increases in female mortality progressively skewed adult sex ratio in the simulated populations toward males, emulating skewness observed in the wild and reducing long-term population sizes (Figure 1). Steeper declines were observed at higher female mortality values (interaction *Scenario X years*: F_2,29874_ = 45242.6, p < 0.0001; LSMEANS statements: basic and scenario 1: t = -158.32, p < 0.0001; basic and scenario 2: t = -952.51, p < 0.0001; scenario 1 and 2: t = 794.21, p < 0.0001; Figure 1).

Population sizes after reinforcements with a low number of individuals (4.06 birds/year) significantly differed from the basic scheme without releases (releases in the basic scenario: F_3,396_ = 66.28, p < 0.0001; releases in scenario 1: F_3,396_ = 115.89, p < 0.0001; releases in the scenario 2: F_3,396_ = 1840.80, p < 0.0001). Reinforcements always increased population sizes. Increases were larger when supplementations were skewed toward females than when they were not skewed or skewed toward males (LSMEANS statements: all p < 0.0001; Table 2). In the latter cases, reinforcements increased bird numbers during the first few years, but since females are the limiting demographic factor they did not result in improved population trends in the long run. All recipient populations with skewed sex-ratios showed negative trends that were not completely offset by supplementations, except when releases were large and biased toward females (LSMEANS: all p < 0.0001; Figure 2).

**Table 2:** Estimates of the long-term population trends (βyrs ± SE) expected under the different simulation scenarios.

	No supplementation (I)	Releases of 2 males and 2 females (II)	Releases of 4 males (III)	Releases of 4 females (IV)	LSMEANS statement***
Basic scenario (M:F = 1)	β_yrs_ = -0.00009 ± 9.43E-6	β_yrs_ = 0.00003 ±7.56E-6	β_yrs_ = -0.00013 ± 8.33E-6	β_yrs_ = 0.00015 ± 7.28E-6	I < III < II < IV
	F_1,99_= 81.66, p < 0.0001	F_1,99_= 11.56, p = 0.0010	F_1,99_= 230.29, p < 0.0001	F_1,99_= 414.49, p < 0.0001	
Scenario 1 (M:F = 1.23)	β_yrs_ = -0.00364 ±1.50E-5	β_yrs_ = -0.00273 ±1.40E-5	β_yrs_ = -0.00353 ±1.50E-5	β_yrs_ = -0.00238 ±1.50E-5	I < III < II < IV
	F_1,99_= 57764.4, p < 0.0001	F_1,99_= 37821.6, p < 0.0001	F_1,99_= 57190.1, p < 0.0001	F_1,99_= 25684.5, p < 0.0001	
Scenario 2 (M:F = 4)	β_yrs_ = -0.02174 ± 3.7E-5	β_yrs_ = -0.01388 ± 3.2E-5	β_yrs_ = -0.01501 ±2.9E-5	β_yrs_ = -0.01285 ± 3.4E-5	I < III < II < IV
	F_1,99_= 336868, p < 0.0001	F_1,99_= 192847, p < 0.0001	F_1,99_= 263385, p < 0.0001	F_1,99_= 141674, p < 0.0001	

LSMEANS statement orders significantly different βyrs. *** all p-values < 0.0001

Larger reinforcement schemes (10 and 20 birds/year over 100 yrs) significantly improved population sizes (interaction between *Scenario X years*: all p < 0.0001). Releasing females was always more effective (i.e., produced a larger increase in population size; Figure 2) than realising either males and females or just males (LSMEANS: all p < 0.0001; Table 2).

Finally, as would be expected, when the population modelling began with smaller populations (300 or 30 individuals), the effect of releasing more birds per year is more important than when starting with 3000 individuals, with populations even increasing in some cases (Figure S1 in File S1). However, trends are similar, and releasing more females, or the same number of each sex, are always better than releasing only males or no birds at all (Figure S1, Table S1 in File S1).

## Discussion

As expected our findings showed that the number of individuals released and the duration of RorR programs are key factors influencing the population demography of a species subjected to supplementations (see also 4). More importantly, we showed that the existence of a male-biased sex ratio increases the probability of extinction [25]. Releasing birds may reduce the rate of population decrease; however, results are improved when taking into account both the sex ratio of the recipient populations and the sex ratio of the released stocks. Whereas releasing both sexes improved population numbers in the long-term, programs releasing a similar number of birds but with a female-biased sex ratio could be more cost-effective. To generalize, our results indicate that the effectiveness of RorR programs, especially for dimorphic species, can be improved by including new considerations such as the sex ratio. If such a parameter is not considered, conservation programs may be less effective or even detrimental to the species under management. This emphasizes the importance of continual guidance and monitoring of the well-intentioned attempts to conserve a species.

Many South American countries are releasing captive-reared Andean condors into the wild to facilitate the recovery of their populations [7]. However, the information available suggests that these RorR programs do not take into account the implications of the sex ratio of these releases, which tend to be male-biased [23,26]. These biases in reinforcements across Andean condor conservation projects may increase the skewness in the sex ratio of wild local populations and, consequently, reduce the probability of success of those conservation projects [27,28]).

It should be noted that our results are more qualitative than quantitative as our projections are based on a theoretical mortality rate. However this value of mortality allowed us to model the different scenarios starting from a stable population, and to show the changing trends when supplementations of different sex-ratios are considered. Moreover, the value used as a surrogate mortality rate is from a similar species and does not differ greatly from that proposed for a specific condor population (see 20). Our PVAs should be viewed as comparative trends for populations differing in sex-ratios and subject to reintroduction programs, also differing in the sex-ratio of the releases. Therefore, it is vital that RorR programs consider the actual impact of the releases on wild populations, as a precautionary principle [2]. This is particularly important for condors considering their sensitivity to small changes in the demographic structure of populations [18].

Decisions on which birds to release rest on many factors such as, for example, genetic information of individuals and considerations of health and behaviour of available birds [1]. However, the random outcomes of captive breeding pairs at breeding facilities may influence the availability of birds for release. Random sampling of sex ratios may be appropriate in RorR programmes for monomorphic vultures [5], but this is not the case for a dimorphic species such as the Andean condor. In general, the number of birds and the duration of the releases are important factors in determining the success of the RorR programs ( [4] and our results). However, for dimorphic species it may be crucial to seriously consider the sex ratio of releases and of the wild population (considering the possibility of sex-biased mortality in the wild) as key factors when designing RorR strategies.

Population models for a large, male-skewed population showed long periods of negative trends (≥100 years) even under RorR scenarios, suggesting that efforts are useless unless drivers of population declines are overcome or at least reduced. The models highlight that RorR projects for these types of species must be carried out over long time periods, making them a very expensive conservation approach [2]. Thus, it is advisable that conservation strategies should start by focusing on improving female survival, which can be strongly dependent on sex-specific habitat selection [6,11,12], before attempting to compensate for high mortality rates through releases [25]. If RorR programs are considered, and bearing in mind that it is important to properly assess their effectiveness for each case [3,5], our results advocate an important role for the proportion of sexes in the releases. Conservationists and managers working in RorR programs should apply this type of evidence-based conservation in designing their programs [29]. Working in constant two-way communication with scientists would greatly improve the success of the RorR programs.

## Supporting Information

File S1 **Figure S1.** Projections of Andean condors population size over time beginning at three different population sizes (3000, 300 and 30 individuals; basic scenarios: grey lines) and under different reinforcement schemes (supplementation with 4 females: blue lines, 4 males: green lines, 2 males and 2 females: purple lines). M:F, proportion of males to females. **Table S1.** Details of the LSMEANS comparisons by pairs between the different scenarios (reinforcement scheme I: no supplementation; II: releases of 2 males and 2 females; III: releases of 4 males; IV: releases of 4 females) starting from populations of 300 and 30 individuals (see the comparisons for 3000 individuals in the main text Table 2). N: population size; M:F, proportion of males to females.
(DOC)
