# Supplementary material for: Population Sex Ratios: Another Consideration in the Reintroduction – Reinforcement Debate?
Source: PLoS One. 2013 Sep 26;8(9):e75821. doi: 10.1371/journal.pone.0075821 (PMC3784389; doi:10.1371/journal.pone.0075821)
Supplement: File S1 — Figure S1. Projections of Andean condors population size over time beginning at three different population sizes (3000, 300 and 30 individuals; basic scenarios: grey lines) and under different reinforcement schemes (supplementation with 4 females: blue lines, 4 males: green lines, 2 males and 2 females: purple lines). M:F, proportion of males to females. Table S1. Details of the LSMEANS comparisons by pairs between the different scenarios (reinforcement scheme I: no supplementation; II: releases of 2 males and 2 females; III: releases of 4 males; IV: releases of 4 females) starting from populations of 300 and 30 individuals (see the comparisons for 3000 individuals in the main text Table 2). N: population size; M:F, proportion of males to females. (DOC) [file pone.0075821.s001.doc]

Supplementary material for:

**Population Sex Ratios: Another Consideration in the Reintroduction –**

**Reinforcement Debate?**

Sergio A. Lambertucci^1^*, Martina Carrete^2^, Karina L. Speziale^1^, Fernando Hiraldo^3^ and José Antonio Donázar^3^

**Figure S1.** Projections of Andean condors population size over time beginning at three different population sizes (3000, 300 and 30 individuals; basic scenarios: grey lines) and under different reinforcement schemes (supplementation with 4 females: blue lines, 4 males: green lines, 2 males and 2 females: purple lines). M:F, proportion of males to females.


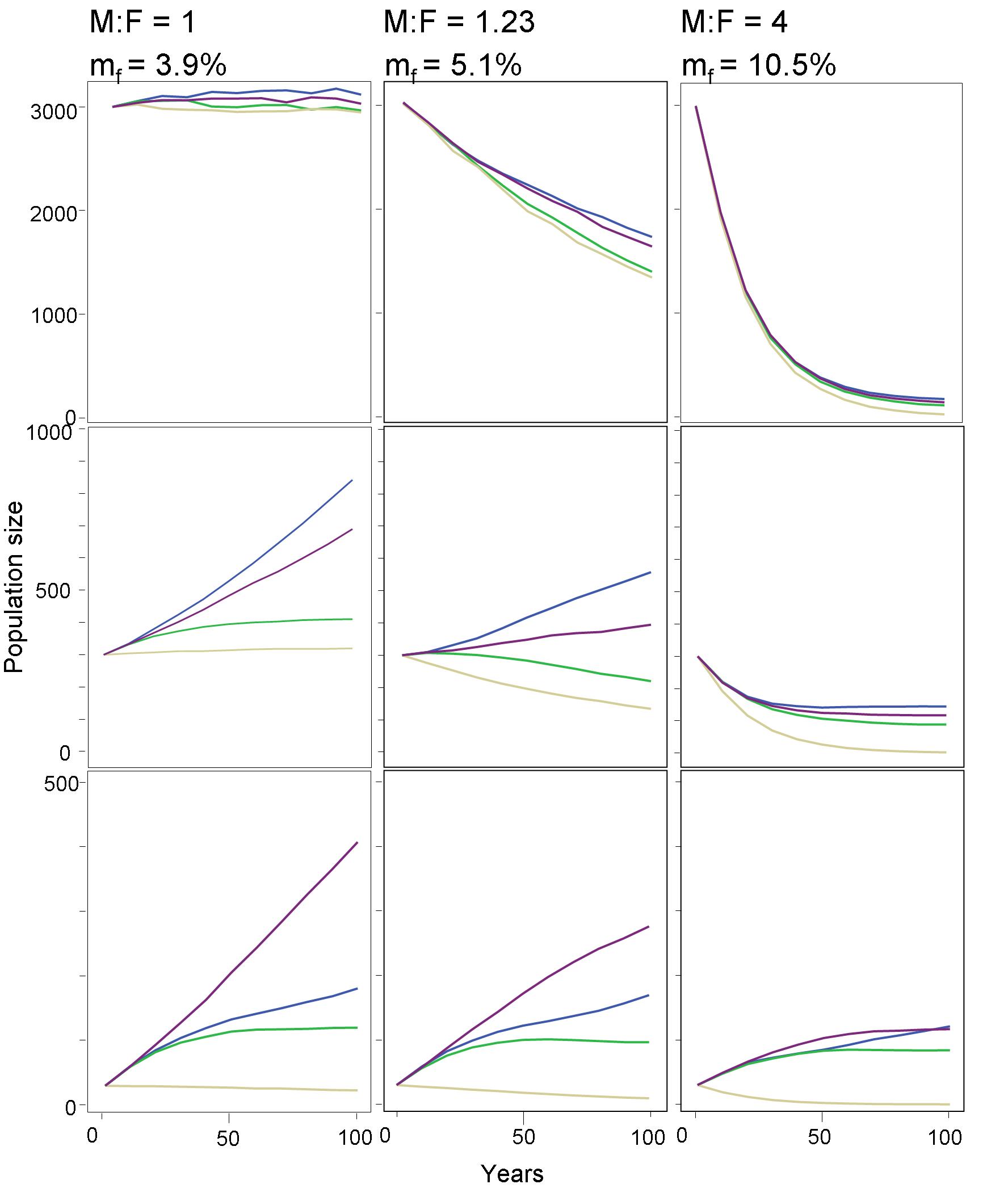


**Table S1.** Details of the LSMEANS comparisons by pairs between the different scenarios (reinforcement scheme I: no supplementation; II: releases of 2 males and 2 females; III: releases of 4 males; IV: releases of 4 females) starting from populations of 300 and 30 individuals (see the comparisons for 3000 individuals in the main text Table 2). N: population size; M:F, proportion of males to females.

| Starting population size | Sex ratio of recipient population | Reinforcement scheme | | | |
| --- | --- | --- | --- | --- | --- |
| **N=300** | **M:F=4** |  | II | III | IV |
|  |  | I | <.0001 | <.0001 | <.0001 |
|  |  | II |  | <.0001 | 0.2987 |
|  |  | III |  |  | <.0001 |
|  | **M:F=1.23** |  | II | III | IV |
|  |  | I | 0.0537 | <.0001 | <.0001 |
|  |  | II |  | <.0001 | <.0001 |
|  |  | III |  |  | <.0001 |
|  | **M:F=1** |  | II | III | IV |
|  |  | I | <.0001 | <.0001 | <.0001 |
|  |  | II |  | <.0001 | 0.0079 |
|  |  | III |  |  | <.0001 |
| **N=30** | **M:F=1** |  | II | III | IV |
|  |  | I | <.0001 | <.0001 | <.0001 |
|  |  | II |  | 0.3372 | <.0001 |
|  |  | III |  |  | <.0001 |
|  | **M:F=4** |  | II | III | IV |
|  |  | I | <.0001 | <.0001 | <.0001 |
|  |  | II |  | <.0001 | 0.0257 |
|  |  | III |  |  | 0.0517 |
|  | **M:F=1.23** |  | II | III | IV |
|  |  | I | <.0001 | <.0001 | <.0001 |
|  |  | II |  | <.0001 | <.0001 |
|  |  | III |  |  | 0.0002 |
